# Expanding management strategies for cervical precancerous lesions in resource-limited settings: insights from a training center in a district hospital in Ghana

**DOI:** 10.1186/s12905-024-03263-0

**Published:** 2024-07-27

**Authors:** Kofi Effah, Ethel Tekpor, Comfort Mawusi Wormenor, Samuel Adolf Bosoka, Maxwell Afetor, Anita Edinam Dugbazah, Stephen Danyo, Esu Aku Catherine Morkli, Georgina Tay, Bernard Hayford Atuguba, Elorm Kpofo-Tetteh, Chrysantus Kubio, Nana Owusu Mensah Essel

**Affiliations:** 1 Cervical Cancer Prevention and Training Centre, Catholic Hospital, Battor, Volta Region Ghana; 2 Department of Obstetrics and Gynecology, Catholic Hospital, Battor, Volta Region Ghana; 3 Disease Surveillance Unit, Volta Regional Health Directorate, Ghana Health Service, Ho, Volta Region Ghana; 4 Department of Epidemiology and Biostatistics, Fred N. Binka School of Public Health, University of Health and Allied Sciences, Ho, Volta Region Ghana; 5 Ho Polyclinic, Ghana Health Service, Ho, Volta Region Ghana; 6 Department of Obstetrics and Gynecology, Korle Bu Teaching Hospital, Accra, Ghana; 7 Volta Regional Health Directorate, Ghana Health Service, Ho, Volta Region Ghana; 8 Department of Emergency Medicine, College of Health Sciences, Faculty of Medicine and Dentistry, University of Alberta, 730 University Terrace, Edmonton, AB T6G 2T4 Canada

**Keywords:** Cervical precancer, Cervical intraepithelial neoplasia, Thermal ablation, Visual inspection with acetic acid, Loop electrosurgical excision procedure, Cold knife conization

## Abstract

**Background:**

Cervical cancer continues to disproportionately burden women in low/middle-income countries like Ghana. We examined treatment patterns and histopathological outcomes among women screened using visual inspection with acetic acid (VIA) and/or mobile colposcopy who subsequently underwent thermal ablation, large loop excision of the transformation zone (LLETZ), or cold knife conization at the Cervical Cancer Prevention and Training Centre, Battor. We also assessed the prevalence of cervical intraepithelial neoplasia 2+ (CIN2+) or micro-invasive disease and their associated factors for women who underwent excisional treatments. The treatment choices for cervical precancerous lesions suitable for resource-limited settings have also been described from the perspective of a center that manages a heterogenous population.

**Methods:**

We conducted an analysis of secondary data collected between June 2016 and June 2023 among women with positive findings on VIA or mobile colposcopy who subsequently underwent thermal ablation or large loop excision of the transformation zone (LLETZ). The prevalence of histopathology outcomes, including no dysplasia, CIN1 − 3, and micro-invasive disease, were estimated with 95% confidence intervals (CIs). Factors associated with histopathological findings were modeled using multinomial logistic regression.

**Results:**

For the study period, 14 (10.6%) of the total 132 participants underwent cervical lesion treatment at outreach locations, all via thermal ablation. The remaining 118 (89.4%) were treated at the Catholic Hospital, Battor using LLETZ (*n* = 66, 55.9%), thermal ablation (*n* = 51, 43.2%), and cold knife conization (*n* = 1, 0.9%). Among 65 women with histopathology reports, the most frequent histopathological finding was no dysplasia (47.7%; 95% CI, 35.1 − 60.5), followed by CIN2 and CIN3 (20.0%; 95% CI, 11.1 − 31.8 each), CIN1 (7.7%; 95% CI, 2.5 − 17.0) and micro-invasion (4.6%; 95% CI, 1.0 − 12.9). Those with micro-invasive disease were significantly older than those with CIN1, CIN2, and CIN3 (*p* = 0.036, 0.022, 0.009, respectively), but not significantly older than those who showed no dysplasia (*p* = 0.088). For each unit increase in age, the likelihood of CIN3 was relatively significantly reduced compared to no dysplasia (crude relative risk ratio [RRR] = 0.93; 95% CI, 0.86 − 0.99). This association was neither observed with the remaining histopathological groups nor for parity and persisted after controlling for parity (adjusted RRR = 0.92; 95% CI, 0.85 − 0.99; *p* = 0.025).

**Conclusion:**

This paper largely demonstrates treatment options available to women and practitioners in LMICs. The high combined prevalence of high-grade precancerous lesions and micro-invasive disease underscores the need to increase cervical cancer awareness that would enhance screening attendance and hasten efforts at moving from opportunistic to organized screening in Ghana. This will enhance early cervical lesion detection and treatment, while simultaneously re-evaluating and cutting down on unnecessary treatment.

## Background

Cervical cancer is the fourth most common malignancy among women globally and resulted in approximately 660,000 incident cases and 350,000 deaths worldwide in 2022 [1]. It continues to disproportionately burden women in low (middle) income countries (LMICs) [2–4] like Ghana, where it is the second most common malignancy among females and was estimated to have accounted for 19.2% of all newly diagnosed cancer cases and 10.1% of deaths due to cancer in 2022 [5]. Vaccination against human papillomavirus (HPV) and early detection via cervical precancer screening, followed by prompt treatment remain the mainstay approach to preventing cervical cancer. Improvements in cervical cancer prevention in high-income settings have been ascribed mostly to the use of cytologic methods to detect preinvasive lesions. In contrast, the relatively low success of campaigns and programs seen in LMICs has been mostly attributed to low population coverage, poor access to (and quality of) cytology, inadequate follow-up of screen positives, and unavailability of effective treatment options [6].

The World Health Organization (WHO) has called for the worldwide elimination of cervical cancer with intermediate objectives of achieving 90% vaccination of target females, screening 70% of women in their mid-adult years, and effectively treating 90% of women requiring treatment for precancer and cancer [7]. Among these pillars, achieving 90% treatment may be the most challenging, since it inherently requires accurate identification of precancerous lesions among all screen positives. Effective management of screen-positive women comprises two main steps: deciding which women to treat and treating them correctly. Currently, both these decision-making and treatment steps tend to be crude and not sufficiently backed by performance data [8].

Major resource investments necessary for training and enhancing the capabilities of multi-stage cervical screening programs often serve as a barrier to implementation in many low-resource settings. Likewise, delivering high-quality excisional treatment requires substantial infrastructure, a proficient and adequately trained workforce, and continuous quality assurance [9]. A significant challenge posed by established screening programs is the need for multiple visits for screening, colposcopy, treatment, and follow-up. Each stage carries the risk of losing women along the care continuum, potentially allowing undetected precancerous lesions to advance to cancer. This loss to follow-up is especially worrying in LMICs, where women have constant domestic duties and may have to travel long distances to healthcare centers, entailing substantial transportation expenses and lost wages. Moreover, reaching them to pass along information on screening outcomes and schedule appointments can prove difficult.

In high-resource settings, risk-based treatment guidelines assist practitioners choose whether to implement immediate treatment, colposcopic referral, or reassurance [8]. While the scientific and clinical bases for treatment should be the same for high- and low-resource settings, affordability and practicality serve as barriers to repetition in the latter setting. Screen and treat approaches in which all screen-positive women undergo treatment are time- and cost-efficient in LMICs [10]; however, they are subject to high overtreatment rates. The optimum approach to managing cervical intraepithelial neoplasia (CIN) in a screen and treat context utilizing visual inspection with acetic acid (VIA) as the primary screening tool is controversial. In Ghana, there has been recent advocacy for a concurrent approach [combined VIA/colposcopy + high-risk HPV (hr-HPV) testing] [11] and tritesting (combined VIA/colposcopy + hr-HPV testing + Pap smear) [12], each in a single visit. As of the time of this report, formal recommendations in this aspect for LMICs (with varying national-level policies) are pending from organizations like the WHO.

In this report, we primarily aimed to share our treatment approaches and report outcomes of women diagnosed with cervical precancerous lesions screened on outreaches or in-clinic at a training center in a district hospital in Ghana between June 2016 and June 2023. Drawing from practice in a setting where cervical precancer screening is largely opportunistic and there is no national policy or organized program for screening, we describe treatment choices for cervical precancerous lesions suitable for resource-limited settings that would be useful for practitioners in similar contexts. Among women who underwent excisional biopsy followed by histopathology, our secondary aim was to estimate the prevalence of CIN2 + disease on excisional specimens and to examine their associated factors.

## Methods

### Study design

We conducted a secondary data analysis with the dual aim of examining treatment patterns and histopathological outcomes among women who presented to the outpatient clinic of the Cervical Cancer Prevention and Training Centre (CCPTC), Catholic Hospital, Battor or on outreaches for cervical precancer screening over a 7-year period (June 2016 to June 2023). Specifically for women who underwent excisional treatments due to positive findings on VIA and/or mobile colposcopy and/or high-grade squamous intraepithelial lesions (HSIL) on cytology, we provided prevalence estimates of CIN2, CIN3, or micro-invasive disease in excision specimens sent for histopathology and examined factors associated with these findings.

### Ethical considerations

The study complied with the Declaration of Helsinki (1964) and its later amendments. Verbal informed consent was sought from the women prior to questionnaire administration, sample collection, and cervical screening. The consent procedure was approved by the Ethical Review Committee of the Catholic Hospital, Battor (approval no. CHB-ERC-002/01/24), which also permitted the researchers to publish the findings of this study.

### Study participants and inclusion/exclusion criteria

Our study included all 132 otherwise asymptomatic women who showed abnormal cervical lesions upon presenting (or referral) for primary cervical screening. The inclusion criteria were all women aged ≥ 25 years who were not pregnant, had an intact uterus and cervix, and were not menstruating. Despite being younger than the recommended cut-off age of 25 years, a few women were also screened on account of being parous and were included in this study if they showed positive findings on VIA or EVA mobile colposcopy. On the other hand, we excluded women who were seriously ill and those who showed lesions suggestive of invasive cervical cancer at screening.

### Study variables and outcomes

We collected details such as the setting and type of treatment administered, as well as sociodemographic characteristics, including age, parity, age at menarche, marital status, religion, and highest education level. Clinical details included past/current contraceptive use, self-reported (or clinically-confirmed) HIV status, smoking, and prior precancer screening. We also recorded details seen on gross genital inspection, the transformation zone (TZ) type seen, and outcomes on each screening method [hr-HPV DNA testing, visual inspection procedures (VIA and/or EVA mobile colposcopy), and cytology (Pap smear)]. Among women who underwent excision treatments and had specimens submitted for histopathology, the outcomes of interest were findings of no precancer (no dysplasia), precancerous lesions (CIN1, CIN2, or CIN3), and micro-invasive disease.

### Data collection and management

After obtaining verbal informed consent, a structured questionnaire was used to capture the data of the participants at the time of screening. The study data were captured and managed via REDCap version 11.0.0 (Vanderbilt University, Nashville, TN, USA) [13] and stored in local secure databases. Prior to the analysis, the data were queried, extracted, and manually crosschecked to ensure accuracy. When histopathological results were obtained, they were matched with the patients using unique identification numbers. The data were then de-identified before transmission for analysis to ensure the privacy and anonymity of the participants.

### Screening procedures

During the study period, cervical precancer screening was performed mainly using hr-HPV-based screening, VIA, cytology (Pap smear), and Enhanced Visual Assessment (EVA) mobile colposcopy. For many women, a concurrent [11] approach (*n* = 66) [hr-HPV testing + a visual inspection method (VIA and/or EVA mobile colposcopy) in the same setting] was used, while for *n* = 60 women, standalone testing (hr-HPV testing alone, cytology alone, or VIA / EVA mobile colposcopy alone) was performed. For a minority of women (*n* = 6), tritesting [hr-HPV testing + a visual inspection method (VIA and/or EVA mobile colposcopy) + cytology in the same setting] [12] was performed.

#### Visual inspection procedures and cervical specimen collection for cytology and hr-HPV DNA testing

Trained nurses conducted cervical screening with the woman lying in a dorsal lithotomy position, using a sterile vaginal speculum to expose the cervix. Liquid-based cytology samples were collected using a Cervex-Brush^®^ and fixed with PreservCyt, while conventional cytology samples were obtained with an Ayre spatula and cytobrush and fixed immediately with 92% alcohol. Liquid-based cytology samples were divided, with a portion submitted for HPV testing. The choice between conventional and liquid-based cytology depended on kit availability, with a preference for the latter. Samples were sent to an external pathologist 100 km away, as the Catholic Hospital, Battor, does not have an in-house cytotechnologist; the results were made available within one week.

VIA and/or EVA mobile colposcopy [using the EVA system 3.0 (MobileODT, Tel Aviv, Israel)] were also performed by trained nurses. The VIA procedure involved examination of the cervix for abnormalities under adequate lighting after applying 5% acetic acid with a waiting period of 90–120 s. Colposcopy findings were documented using the International Federation of Cervical Pathology and Colposcopy (IFCPC) terminology, noting adequacy, TZ type, and the presence of significant cervical lesions [14]. VIA results were categorized as ‘positive’ if aceto-white lesions were observed at the TZ. The TZ types observed at colposcopy or VIA were also reported according to the IFCPC 2011 criteria [14, 15].

#### Laboratory processing of cervical specimens and HPV DNA assays

Depending on the testing platform in use at the CCPTC when a woman presented for screening, cervical samples obtained via the aforementioned procedures underwent processing and analysis using one of four systems: the *care*HPV system (Qiagen GmBH, Hilden, Germany), GeneXpert HPV system (Cepheid, Sunnyvale, CA, USA), AmpFire HPV DNA test platform (Atila BioSystems, Inc., Mountain View, USA), or the MA-6000 HPV assay system (Sansure Biotech Inc., Hunan, China). All tests were conducted at the central laboratory of Catholic Hospital, Battor, following the manufacturers’ instructions and as detailed elsewhere [16–21]. *care*HPV tests were run using cervical specimens in *care*HPV collection medium, GeneXpert assays utilized cervical specimens in PreservCyt or ThinPrep liquid cytology specimens, while AmpFire and MA-6000 assays used genital swabs (dry brushes, swabs, or PreservCyt/ThinPrep). In terms of genotype classification, *care*HPV collectively (non-specifically) identifies HPV16/18/31/33/35/39/45/51/52/56/58/59/66/68. The GeneXpert HPV assay gives results from six separate channels: sample adequacy control, P1 − HPV16, P2 − HPV18/45, P3 − HPV31/33/35/52/58, P4 − HPV51/59, and P5 − HPV39/68/56/66. The AmpFire and MA-6000 tests were performed using semi-quantitative modules, which specifically identify HPV16 and HPV18, and collectively identify HPV31/33/35/39/45/51/52/53/56/58/59/66/68 as *other* high-risk HPV genotypes.

### General eligibility criteria for treatments: thermal ablation, LLETZ, and cold knife conization

For VIA/EVA-positive-screened women, thermal ablation or large loop excision of the TZ (LLETZ) was commonly offered. All LLETZ and cold knife conization procedures were performed by a specialist gynecologist at the CCPTC, Catholic Hospital, Battor. Thermal ablation, on the other hand, was commonly administered by a trained nurse, midwife, or medical officer.

As per the 2019 WHO Guidelines for the Use of Thermal Ablation for Cervical Precancer Lesions [22], screen-positive women who showed no suspicion of invasive disease or glandular lesions (adenocarcinoma) were eligible for thermal ablation if the entire TZ was fully visible and did not extend to the endocervical canal (type 1 TZ). For type 2 TZ, the tip of the probe should be able to reach the upper border of the TZ and achieve complete ablation of the squamocolumnar junction [15]. The Liger Medical thermal ablator (Liger/Cure Medical LLC, Utah, USA) was used; our protocol involved probe application at 100 °C for 45 s. To be considered eligible for LLETZ, there should also be no evidence of frank cancer like large fungating or ulcerating lesions. For these, biopsies were taken from the tumors. LLETZ biopsies were performed by removing the entire lesion or TZ with loops selected based on the size of the lesion or TZ. The loops were connected to electrosurgical or diathermy units and cones of the cervix were taken out by the heated loops, usually in a single sweep. The cervical specimens were submitted to a pathologist for morphological examination. As per standard operating procedures, excised specimens were stored in 10% buffered formalin before transporting them to a pathologist. After processing, the specimen was embedded in paraffin wax, sectioned into 5-micron slices, and stained with hematoxylin-eosin. The pathologist looked at several slides of each specimen with an Olympus microscope to make the final pathological anatomical diagnosis of the cervical lesion. For one participant, cold knife conization was performed under spinal anesthesia since the lesion extended into the squamocolumnar junction by more than 5 mm. Cold knife conization involved the excision of a cone of the cervix with the lesion under subarachnoid block with a scalpel and securing hemostasis on the cervix with ball diathermy. For histopathological examination, multiple sections were made from the excised cone which had been fixed in formalin and embedded in paraffin. Sections with a thickness of 4 μm were cut (12 sections in all) and processed routinely for hematoxylin-eosin staining.

### A shared decision-making approach to treatment selection and follow-up for screen-positives

The cost of treating cervical precancerous lesions is generally not covered by the National Health Insurance Scheme or government support in Ghana. A shared decision-making approach was used in selecting treatment methods and following up screen positives in our setting, after thorough discussion with the women and taking into consideration the clinical picture and their economic circumstances. While excisional treatment may be the gold standard [23] due to its ability to arrive at a histopathological/morphological diagnosis, it is not always possible or available to women in our setting primarily due to the unavailability of histopathology services. Even if available, they are generally considered expensive and women (who have to pay out-of-pocket) often cannot afford it. Excisional treatment also requires anesthesia, further increasing associated costs. Thus, ablative treatment was often advantageous in that it is generally cheaper and does not require anesthesia. In addition, it can be performed by middle and low-cadre staff and can be administered on-site. For women who could afford the cost of histopathology, a biopsy was taken and treatment was deferred until the histopathology report came in. In certain instances where women could afford the cost of biopsy (but could temporarily/partially not afford histopathology), our experience was that it was better to administer the treatment and keep the cervical specimen in formalin while the women looked for money for histopathology to reduce the risk of progression (to cancer) or loss to follow-up.

Generally, after treatment (thermal ablation/LLETZ), women were asked to abstain from intercourse for 4 weeks. Where this was not possible, they were advised to use condoms during sex. Persistent hr-HPV infection is strongly linked with lesion recurrence [24] and the risk of CIN2 + lesion recurrence increases in proportion to the duration of hr-HPV persistence up to 1 year [25, 26]. Thus, if there were no complications (such as offensive vaginal discharge or bleeding per vaginam), women were typically followed up after 1, 6, and 12 months. At 6 or 12 months, women who could afford to do so underwent tritesting (involving hr-HPV DNA testing, cytology, and colposcopy) as a ‘test of cure’. If all three tests yielded negative results, the woman could join routine screening. In the past, women were also offered HPV vaccination at this time to protect them against HPV infection; however, recent evidence has shown that the best time to administer vaccination is just before or after treatment [27]. This is particularly important for women in polygamous relations who may go back to the same risk settings and acquire HPV infection again before the ‘test of cure’ can be performed at 6 or 12 months.

### Statistical methods

We evaluated the distributions of continuous and discrete data such as age and parity both visually using histograms and the Shapiro − Wilk test. Nominal characteristics such as highest education level, marital status, and types of treatment administered (LLETZ, thermal ablation, and cold knife conization) are reported as counts and percentages. Symmetrically distributed continuous/discrete data are reported as means with their standard deviations (SDs) while those with non-normal distributions are reported as medians with interquartile ranges (IQRs). Although a much smaller number of women (*n* = 14) were screened in the outreach setting, they were found to significantly differ from those screened at the Battor Clinic (*n* = 118) in terms of age, age at menarche, and number of lifetime pregnancies (Table 1). Thus, for all characteristics and outcomes observed at initial screening (apart from findings on cytology which was only carried out in-clinic), we present overall estimates as well as setting-specific estimates. The prevalence of no dysplasia, CIN1, CIN2, CIN3, and micro-invasive disease as outcomes of histopathology are reported in rate form with 95% Clopper − Pearson confidence intervals (CIs) and were compared by age and parity using the Wilcoxon rank-sum test between pairs of histopathology categories and the Kruskal − Wallis *H* test across. We then fit unadjusted and adjusted polytomous (multinomial) logistic regression models to test the hypotheses that the severity of histopathology findings (no dysplasia, CIN1 − 3, or micro-invasive disease) varies by age and parity. In each regression equation, the outcome was modeled as a 4-level categorical variable with the exposure variables age (continuous) and parity (discrete). The size and directionality of the effect estimates are reported as crude and adjusted relative risk ratios (RRRs) with 95% CIs. All statistical analyses were performed using Stata version 18.5 (StataCorp LLC, College Station, TX, USA). Each null hypothesis was rejected at a two-tailed alpha level of 5%.

## Results

### Sociodemographic and clinical characteristics of the study participants

A total of 132 women met the inclusion criteria and were included in the study. Treatment was administered in an outreach setting for 14 of these women (10.6%) and for the remaining 118 (89.4%) at the clinic of the Catholic Hospital, Battor (Table 1). Overall, the participants had a median age of 35 (IQR: 27, 42) years and a median parity of 1 (IQR: 0, 2). A majority, *n* = 79 (59.9%) of the women belonged to the Christian faith. Nine (6.8%) women reported being HIV positive, while the remaining were either HIV negative or did not know their status. About a third (27.5%) had undergone prior precancer screening.

Women screened in the outreach setting were significantly younger than those screened in the clinic (*p*-value = 0.008), experienced menarche at a significantly younger age (*p*-value < 0.001), and had experienced fewer pregnancies during their lifetimes (*p*-value = 0.045). They were however statistically similar in terms of distribution for the remaining characteristics assessed, including education level, contraceptive use, marital status, income level, and prior cervical precancer screening (Table 1).

**Table 1:** Distribution of sociodemographic and clinical characteristics of screen-positive women who underwent treatment stratified by treatment setting

Characteristic	Treatment setting		*p*-value	Total (*n* = 132)
	Battor clinic (*n* = 118)	Outreach (*n* = 14)		
Age, years; median (IQR)	36.0 (28.0, 43.0)	26.5 (24.0, 36.0)	0.008*	35.0 (27.0, 42.0)
Age group, years; n (%)			0.243	
<30	34 (28.8)	8 (57.1)		42 (31.8)
30 − 39	40 (33.9)	5 (35.7)		45 (34.1)
40 − 49	22 (18.6)	1 (7.1)		23 (17.4)
50 − 59	13 (11.0)	0 (0.0)		13 (9.9)
≥60	9 (7.6)	0 (0.0)		9 (6.8)
Age at menarche, years; mean (SD)	15.5 (2.6)	13.8 (2.0)	< 0.001*	15.3 (2.6)
Marital status, n (%)			0.963	
Single	21 (17.8)	3 (21.4)		24 (18.2)
Married	45 (38.1)	6 (42.9)		51 (38.6)
Cohabiting	26 (22.0)	3 (21.4)		29 (22.0)
Divorced	9 (7.6)	1 (7.1)		10 (7.6)
Widowed	7 (5.9)	1 (7.1)		8 (6.1)
Missing	10 (8.5)	0 (0.0)		10 (7.6)
Number of lifetime pregnancies, median (IQR)	2 (0, 3)	1 (0, 1)	0.045*	1 (0, 3)
Number of children, median (IQR)	1 (0, 2)	0 (0, 1)	0.441	1 (0, 2)
Highest education level, n (%)			0.374	
No formal education	48 (40.7)	9 (64.3)		57 (43.2)
Elementary education	32 (27.1)	1 (7.1)		33 (25.0)
Secondary education	16 (13.6)	2 (14.3)		18 (13.6)
Tertiary education	21 (17.8)	2 (14.3)		23 (17.4)
Missing	1 (0.9)	0 (0.0)		1 (0.8)
Religious faith, n (%)			0.154	
Christian	73 (61.9)	6 (42.9)		79 (59.9)
Islam	13 (11.0)	0 (0.0)		13 (9.9)
African traditional religion	1 (0.9)	0 (0.0)		1 (0.8)
No religion	30 (25.4)	8 (57.1)		38 (28.8)
Missing	1 (0.9)	0 (0.0)		1 (0.8)
Earns an income, n (%)	69 (58.5)	6 (42.9)	0.345	75 (56.8)
Income level^α^, GH¢; n (%)			0.194	
<100	8 (11.6)	1 (16.7)		9 (12.0)
100 − 250	21 (30.4)	2 (33.3		23 (30.7)
250 − 500	7 (10.1)	1 (16.7)		8 (10.7)
>500	25 (36.2)	2 (33.3)		27 (36.0)
Unable to say/missing	8 (11.6)	0 (0.0)		8 (10.7)
Past contraceptive use, n (%)	49 (41.5)	8 (57.1)	0.265	57 (43.2)
Current contraceptive use, n (%)	13 (11.0)	3 (21.4)	0.377	16 (12.1)
HIV status, n (%)			0.595	
Positive	9 (7.6)	0 (0.0)		9 (6.8)
Negative	41 (34.8)	4 (28.6)		45 (34.1)
Unknown/missing	68 (57.6)	10 (71.4)		78 (59.1)
Ever smoked, n (%)	1 (0.9)	0 (0.0)	1.000	1 (0.8)
Prior (pre)cancer screening, n (%)	35 (29.9)	1 (7.1)	0.111	36 (27.5)

HIV, Human Immunodeficiency Virus; SD, standard deviation; IQR, interquartile range. ^α^ Among women who reported earning an income. * Statistically significant

### Characteristics and outcomes observed at initial screening

The overall and setting-specific characteristics and outcomes observed at primary screening among the study participants are shown in Table 2. On gross cervical inspection, *n* = 13 (9.9%) women showed cervical abnormalities. The commonest TZ type seen on VIA or EVA mobile colposcopy was type 3 (*n* = 57, 45.6%), followed by type 2 (*n* = 49, 39.2%). Among women who underwent hr-HPV testing as part of the screening, the prevalence of hr-HPV infection was 66.7% (95% CI, 51.0 − 80.0). The ‘positivity’ rate recorded among women screened using either visual inspection method was 62.4% (95% CI, 53.3 − 70.9), disaggregated as 72.5% (95% CI, 58.3 − 84.1) and 53.3% (95% CI, 42.5 − 63.9) among those screened using VIA and EVA mobile colposcopy, respectively. Women who underwent cytology as part of the initial screening showed a prevalence of 34.8% (95% CI, 16.4 − 57.3) for HSIL, 8.7% (95% CI, 1.1 − 28.0) for ASCUS, and 4.3% (95% CI, 0.1 − 21.9) each for adenocarcinoma and atypical glandular cells (Table 2).

**Table 2:** Overall and setting-specific characteristics and outcomes observed at primary screening in the study

	Estimates		
	Battor clinic (*n* = 118)	Outreach (*n* = 14)	Overall (*n* = 132)
**Gross screening characteristic**			
Abnormal vulval inspection findings, n (%)	6 (5.1)	1 (7.1)	7 (5.3)
Abnormal vaginal inspection findings, n (%)	5 (4.2)	1 (7.1)	6 (4.6)
Missing	5 (4.2)	0 (0.0)	5 (3.8)
Cervical inspection findings, n (%)			
Normal	99 (83.9)	14 (100.0)	113 (85.6)
Abnormal	13 (11.0)	0 (0.0)	13 (9.9)
Missing	6 (5.1)	0 (0.0)	6 (4.6)
TZ type^α^seen at visual inspection (VIA or EVA mobile colposcopy)			
1	13 (11.7)	4 (28.6)	17 (13.6)
2	40 (36.0)	9 (64.3)	49 (39.2)
3	57 (51.4)	0 (0.0)	57 (45.6)
Missing	1 (0.9)	1 (7.1)	2 (1.6)
**Screening outcome (prevalence estimates)**			
hr-HPV positive*, % (95% CI)	66.7 (51.0 − 80.0)	-	66.7 (51.0 − 80.0)
Visual inspection positive**, % (95% CI)	58.6 (48.8 − 67.8)	92.9 (66.1 − 99.8)	62.4 (53.3 − 70.9)
VIA ‘positive’^β^, % (95% CI)	65.0 (48.3 − 79.4)	100 (71.5 − 100.0)	72.5 (58.3 − 84.1)
EVA ‘positive’^γ^, % (95% CI)	51.8 (40.7 − 62.7)	80.0 (28.4 − 99.5)	53.3 (42.5 − 63.9)
Cytology finding^δ^, % (95% CI)			
Adenocarcinoma	4.3 (0.1 − 21.9)	-	4.3 (0.1 − 21.9)
ASCUS	8.7 (1.1 − 28.0)	-	8.7 (1.1 − 28.0)
Atypical glandular cells	4.3 (0.1 − 21.9)	-	4.3 (0.1 − 21.9)
HSIL	34.8 (16.4 − 57.3)	-	34.8 (16.4 − 57.3)
NILM	47.8 (26.8 − 69.4)	-	47.8 (26.8 − 69.4)

hr-HPV, high-risk human papillomavirus; TZ, transformation zone; VIA, visual inspection with acetic acid; CI, confidence interval; ASCUS, atypical squamous cells of undetermined significance

^α^ Transformation zone types among 125 women who underwent VIA and/or colposcopy

TZ1: The entire circumference of the squamocolumnar junction is visible; fully ectocervical

TZ2: The entire circumference of the squamocolumnar junction is visible; partly or fully endocervical

TZ3: The entire circumference of the squamocolumnar junction is not visible; partly or fully endocervical

* Among 30 out of 45 women with valid results for hr-HPV tests (*care*HPV, *n* = 23; GeneXpert, *n* = 4; AmpFire, *n* = 27; MA-6000, *n* = 19; missing test type, *n* = 4; invalid test result, *n* = 1, missing test result, *n* = 31)

** Among 125 women who underwent VIA and/or colposcopy

^β^ Among 51 out of 58 women who had findings on VIA recorded (missing, *n* = 7)

^γ^ Among 90 women who underwent EVA mobile colposcopy

^δ^ Among 23 out of 24 women with satisfactory Pap smears (unsatisfactory Pap smear, *n* = 1)

### Treatments received and prevalence of (pre)cancerous lesions identified on histopathology

Among the 118 (89.4%) women treated at the Catholic Hospital, Battor, the most common procedure was LLETZ (*n* = 66, 55.9%), followed by thermal ablation (*n* = 51, 43.2%) and cold knife conization (*n* = 1, 0.9%). Among women who underwent excision treatment and had histopathology done, the most frequent finding was no dysplasia (47.7%; 95% CI, 35.1 − 60.5), followed by CIN2 and CIN3 (20.0%; 95% CI, 11.1 − 31.8 each). CIN1 was also found in 7.7% (95% CI, 2.5 − 17.0) and micro-invasive disease in 4.6% (95% CI, 1.0 − 12.9) of these women (Table 3).

**Table 3:** Treatments implemented and histopathological outcomes of samples taken during excisional treatment

**Treatment type,** *n* (%)	
Thermal ablation	65 (49.2)
LLETZ	66 (50.0)
Cold knife conization	1 (0.8)
**Histopathological outcomes**	
No dysplasia, % (95% CI)	47.7 (35.1 − 60.5)
CIN1, % (95% CI)	7.7 (2.5 − 17.0)
CIN2, % (95% CI)	20.0 (11.1 − 31.8)
CIN3, % (95% CI)	20.0 (11.1 − 31.8)
Micro-invasive disease, % (95% CI)	4.6 (1.0 − 12.9)

CIN, cervical intraepithelial neoplasia; LLETZ, large loop excision of the transformation zone; CI, confidence interval

### Factors associated with (pre)cancerous cervical lesions in the study

**Fig. 1:**
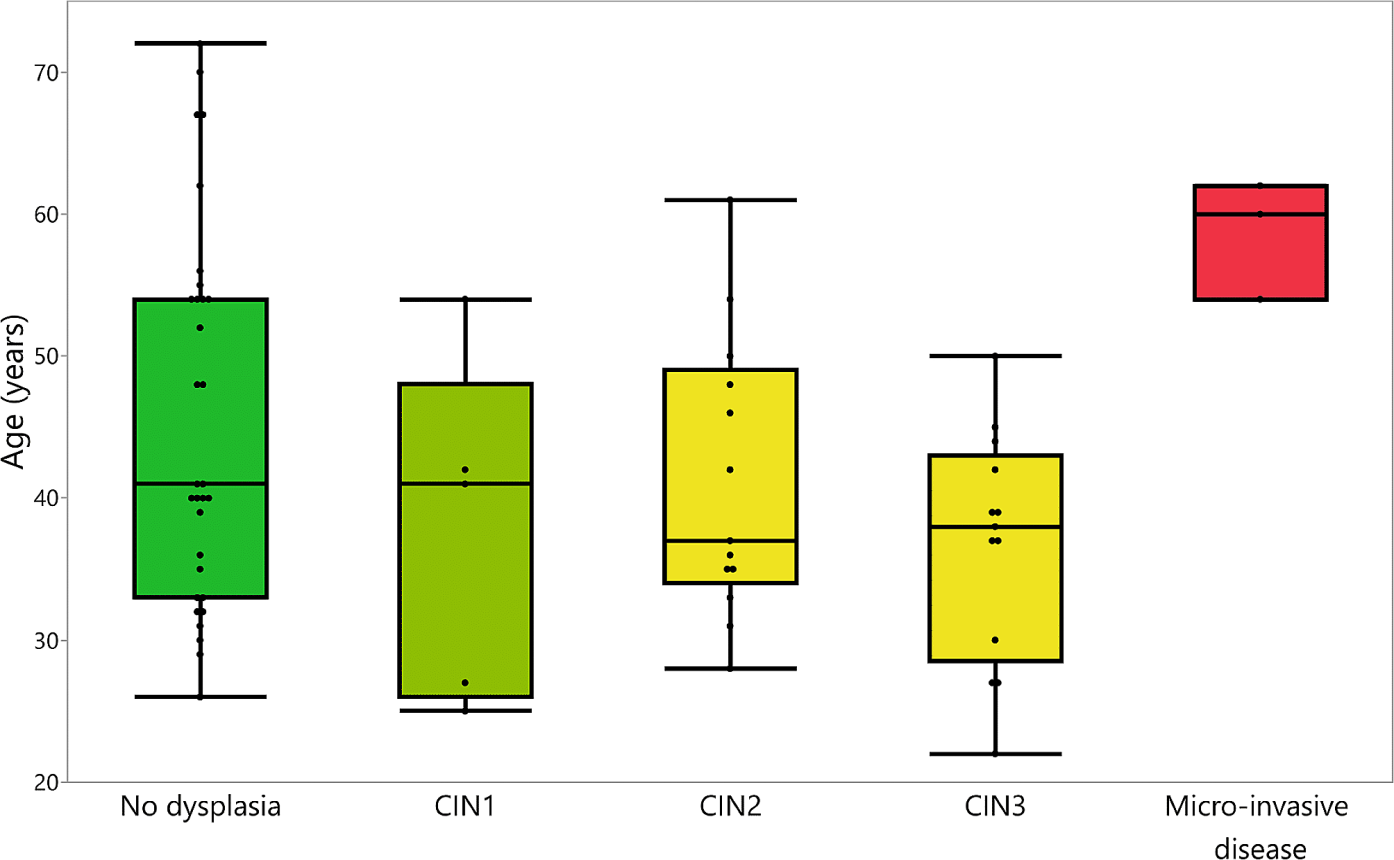
Nested box plots showing the distribution of age stratified by histopathological findings among women who underwent excisional treatments (LLETZ or cold knife conization)

Overall, women who showed micro-invasive disease tended to be significantly older than those with CIN1, CIN2, and CIN3 (Wilcoxon rank-sum *p*-values = 0.036, 0.022, 0.009, respectively), but not significantly older than those who showed no dysplasia (Wilcoxon rank-sum *p*-value = 0.088) on histopathology (Fig. 1). Table 4 shows the results of multinomial logistic regression analysis of factors associated with each positive finding on histopathology compared to no dysplasia. In the unadjusted analysis, each unit increase in age was significantly associated with decreased likelihood of having CIN3 compared to no dysplasia (crude RRR = 0.93; 95% CI, 0.86 − 0.99; *p*-value = 0.030). This association was neither observed with the remaining histopathological groups (CIN1, CIN2, and micro-invasive disease) nor for parity. The association also persisted for CIN3 after controlling for parity (adjusted RRR = 0.92; 95% CI, 0.85 − 0.99; *p*-value = 0.025).

**Table 4:** Exploratory multinomial logistic regression analysis of the association between age and parity for each outcome of histopathology compared to *no dysplasia*

Histopathology result	Factor^α^	Unadjusted models		Adjusted models	
		Crude RRR (95% CI)	*p*-value	Adjusted RRR (95% CI)	*p*-value
**No dysplasia (** ***baseline outcome*** **)**		-	-	-	-
**CIN1**	Age, years	0.94 (0.85 − 1.03)	0.188	0.94 (0.86 − 1.03)	0.216
	Parity	0.45 (0.13 − 1.56)	0.207	0.46 (0.13 − 1.62)	0.225
**CIN2**	Age, years	0.97 (0.91 − 1.03)	0.273	0.97 (0.91 − 1.03)	0.267
	Parity	1.05 (0.65 − 1.68)	0.855	1.07 (0.66 − 1.72)	0.792
**CIN3**	Age, years	0.93 (0.86 − 0.99)	0.030*	0.92 (0.85 − 0.99)	0.025*
	Parity	1.17 (0.74 − 1.83)	0.500	1.28 (0.78 − 2.08)	0.325
**Micro-invasive disease**	Age, years	1.10 (0.98 − 1.23)	0.106	1.09 (0.98 − 1.23)	0.119
	Parity	0.65 (0.19 − 2.19)	0.489	0.71 (0.22 − 2.29)	0.567

CIN, cervical intraepithelial neoplasia; RRR, relative risk ratio; CI, confidence interval

^α^ The factors age and parity are each considered to scale (continuously and discretely, respectively), not categorically

* Statistically significant

## Discussion

There has been a decreasing trend in the cervical cancer burden on a global scale over the past few decades; however, the disease continues to disproportionately constitute a significant public health issue in LMICs like Ghana [28, 29]. Poor prognosis associated with late diagnostic stage, poor knowledge about screening, and poor access to screening in LMICs call for additional considerations especially where there is limited vaccination and screening coverage. In addition, documenting data on histopathological profiles of excisional cervical treatments carried out in institution-based studies, such as ours, is important in unraveling the overall picture of precancerous lesions of the cervix to guide policy making and to serve as a basis for tailored management of patients and follow-up. To the best of our knowledge, this is the first study to examine treatment patterns and histopathological outcomes of cervical precancer screening among otherwise asymptomatic women at an institutional level and in Ghana in general. In this study, a large majority of women who underwent excision treatments showed no dysplasia (47.7%, Table 3). The corresponding prevalence was 7.7% for CIN1, which has a high rate of spontaneous regression within two years (> 85%), with occasional advancement to cancer [30]. However, the commonest precancerous lesions (CIN2 and CIN3, with a combined prevalence of 40%) and micro-invasive disease (prevalence of 4.6%), if left untreated, could progress to frank cervical cancer [31]. The high combined prevalence of precancerous lesions and micro-invasive disease in our study implies the need to increase awareness and hasten efforts at moving from opportunistic to organized screening in Ghana, to enhance early cervical lesion detection and treatment.

Despite the absence of a similar prior study in Ghana with which we could compare our prevalence estimates, our estimated prevalence of 40.0% (95% CI, 28.0 − 52.9) for precancer was higher than reported in a 5-year retrospective cross-sectional review of post-LLETZ cases in Tanzania (30.4%; 95% CI, 25.5 − 35.7) [10], while our estimated prevalence of micro-invasion (4.6%; 95% CI, 1.0 − 12.9) was not too dissimilar (4.0%; 95% CI, 2.1 − 6.7). Our estimates for high-grade lesions and micro-invasion (CIN2+) altogether were similar to those recorded in an Ethiopian cohort following cervical punch biopsy (49.0%; 95% CI, 43.6–54.2) [30].

In both prior studies conducted in Ethiopia and Tanzania [10, 30], age was found to be associated with the occurrence of precancer. The exact higher-likelihood associations observed in Ethiopia and Tanzania were not seen in our study; however, increasing age was found to significantly decrease the likelihood of CIN3 compared to no dysplasia, with/without controlling for parity (adjusted RRR = 0.92, *p*-value = 0.025). Our study may have not been sufficiently powered to detect such differences if they existed, and the association did not reach statistical significance when considering CIN2 + as a binary outcome (odds ratio = 1.33; 95% CI, 0.26 − 6.82 for women aged 30 − 39 vs. ≥50 years). The disparity could additionally have stemmed from the larger sample sizes and the older age demographic in comparison to ours; the mean age in both studies was above 40 years while in our study, the median age was 35 years. Thus, both Ethiopian and Tanzanian studies were more representative of the age bracket within which persistent HPV infections (and thus, more severe histopathological patterns) were more likely.

In our work, a high percentage of those who had histopathology after LLETZ showed no dysplasia (47.7%; 95% CI, 35.1 − 60.5) (Table 3). Several factors may account for this. The criterion for adjudging positivity on VIA or mobile colposcopy is commonly the presence of aceto-whitening, which may be due to immature metaplasia, inflammation, subclinical papillomavirus infection, or CIN. Biopsies for histopathology would have been useful to confirm precancerous (CIN2+) lesions [11]. Similar findings were seen in a study in Tanzania [10] that looked at 329 women who were VIA positive but were not eligible for cryotherapy and had LLETZ. There were 203 (61.7%) patients with benign lesions, including 4 with schistosomiasis and 2 with cervical tuberculosis. Our own work at the CCPTC [32] also showed that quality assurance in cytology reporting is very important as some cytology reports of HSIL revealed no dysplasia after LLETZ. This is especially so when the TZ is type 3, no lesions are seen on the ectocervix on colposcopy, and it is assumed that the high-grade lesion is in the endocervical canal. Also, in low-resource settings where women are likely to be screened few times in their lifetimes, it may be advantageous to use tests with high sensitivity like HPV DNA testing. This will allow limited resources to be used for follow up of women who test positive and may be at a higher risk of developing cervical cancer. The problem here is that this may also cause anxiety in women who persistently test positive for hr-HPV but have no dysplasia. They are at risk of overtreatment. In fact, some of them opt for treatment even when there is no evidence of dysplasia/precancer.

Our center has a cryotherapy unit but only uses it to train health workers. Some trainees only have cryotherapy in their institutions for the treatment of cervical precancer so benefit from this training. While cryotherapy has been used on outreaches in the past, the nurses at the center currently prefer thermal ablation because it is more portable, does not require a cylinder and gases (which makes it difficult to carry along during outreaches), and treatment is shorter with thermal ablation − 40/45 seconds compared to 16 min with the ‘double freeze’ technique for cryotherapy (3 min freeze, 5 min thaw, 3 min freeze, 5 min thaw).

### Strengths and limitations

In this study, we provided the first report of treatments offered and histopathology patterns following excision treatment among otherwise asymptomatic women who presented for screening at a single training institution in Ghana. However, our findings should be interpreted taking into consideration the following limitations. First, after excision, the histopathology reports were generated by a single pathologist; the slides were not double-checked by a second pathologist. Second, our study was a secondary data analysis and was generally restricted to information obtained from the women at screening. This included information about self-reported HIV status, which was generally unknown for a majority of the women and could only be confirmed for a small proportion of them who underwent HIV testing at the time of screening. Third, ensuring that women would adhere to recommendations and return for follow-up after treatment with thermal ablation or LLETZ was a recognized challenge, especially where women lived far from the treatment center or could not afford the cost of follow-up.

## Conclusions

This study primarily draws on documenting histopathological profiles of excisional cervical treatments and demonstrating treatment options available to women with cervical precancerous lesions in LMICs. Our secondary aim of investigating the combined and disaggregated prevalence of CIN2 + lesions was also met, although a large majority of women showed no dysplasia following excisional treatment, implying they did not originally require LLETZ/conization. Nonetheless, although asymptomatic at screening, a significant proportion showed precancerous lesions and micro-invasion. While some association between histopathological pattern and age was also found for CIN3, our study could have been underpowered to test such differences. The high combined prevalence of precancerous lesions and micro-invasive disease further may highlight the need to increase awareness and hasten efforts at increasing screening coverage in Ghana, to enhance early cervical lesion detection and treatment, while simultaneously re-evaluating and cutting down on unnecessary treatment.

## Data Availability

The data that support the findings of this study are available from the Cervical Cancer Prevention and Training Centre (CCPTC), Battor, Ghana, but restrictions apply to the availability of these data. The data are, however, available from the authors upon reasonable request and with permission from the Ethics Review Board, Catholic Hospital, Battor, Ghana.

## Abbreviations

ASCUS: Atypical squamous cells of undetermined significance
CCPTC: Cervical Cancer Prevention and Training Centre
CI: Confidence interval
CIN: Cervical intraepithelial neoplasia
EVA: Enhanced Visual Assessment
hr-HPV: High-risk human papillomavirus
IFCPC: International Federation of Cervical Pathology and Colposcopy
IQR: Interquartile range
LLETZ: Large loop excision of the transformation zone
LMIC: Low- (middle-) income country
RRR: Relative risk ratio
SD: Standard deviation
TZ: Transformation zone
VIA: Visual inspection with acetic acid
WHO: World Health Organization
